# Food Cure

**Published:** 1858-12

**Authors:** 


### FOOD CURE.

THIS Journal aims to show how to maintain health by natural agencies, and by the same means to restore it if lost. It is not pretended that all diseases are cured in this way; but it is very certain that quite a number of ordinary ailments may be removed by the judicious employment of the contents of a well-furnished larder—and with this great advantage, the cures are more permanent, and less liable to return—accomplishing their object without any shock to the system, and without the danger of killing the patient, by mistaking the quantity, or quality, or name of the dose.

Ripe fruits and berries, slightly acid, will remove the ordinary diarrhœas of early summer.

Common rice, parched brown like coffee, and then boiled and eaten in the ordinary way, without any other food, is, with perfect quietude of body, one of the most effective remedies for troublesome looseness of bowels.

Some of the severest forms of that distressing ailment called dysentery, that is, when the bowels pass blood, with constant desire, yet vain efforts to stool, are sometimes entirely cured by the patient eating a heaping tablespoon, at a time, of raw beef, cut up very fine, and repeated at intervals of four hours, until cured, eating and drinking nothing else in the meanwhile.

If a person swallows any poison whatever, or has fallen into convulsions from having overloaded the stomach, an instantaneous remedy, more efficient and applicable in a larger number of cases than any half a dozen medicines we can now think of, is a heaping teaspoon of common salt and as much ground mustard, stirred rapidly in a teacup of water, warm or cold, and swallowed instantly. It is scarcely down before it begins to come up, bringing with it the remaining contents of the stomach; and lest there be any remnant of a poison, however small, let the white of an egg, or a teacup of strong coffee, be swallowed as soon as the stomach is quiet; because these very common articles nullify a larger number of virulent poisons than any medicines in the shops.

In case of scalding or burning the body, immersing the part in cold water gives entire relief, as instantaneously as the lightning. Meanwhile, get some common dry flour, and apply it an inch or two thick on the injured part, the moment it emerges from the water, and keep sprinkling on the flour through anything like a pepper-box cover, so as to put it on evenly. Do nothing else, drink nothing but water, eat nothing, until improvement commences, except some dry bread softened in very weak tea of some kind. Cures of frightful burnings have been performed in this way, as wonderful as they are painless.

Erysipelas, a disease often coming without premonition, and ending fatally in three or four days, is sometimes promptly cured by applying a poultice of raw cranberries pounded, and placed on the part over night.

Insect bites, and even that of a rattlesnake, have passed harmless, by stirring enough of common salt in the yolk of a good egg to make it sufficiently thin for a plaster, to be kept on the bitten parts.

Neuralgia and toothache are sometimes speedily relieved by applying to the wrist a quantity of bruised or grated horse-radish.

Costive bowels have an agreeable remedy in the free use of tomatoes at meals—their seeds acting in the way of the seeds of white mustard or figs, by stimulating the coats of the bowels over which they pass, in their whole state, to increased action.

A remedy of equal efficiency, in the same direction, is cracked wheat—that is, common white wheat grains, broken into two or three pieces, and then boiled until it is as soft as rice, and eaten mainly at two meals of the day, with butter or molasses.

Common sweet cider, boiled down to one-half, makes a most excellent syrup for coughs and colds for children—is pleasant to the taste, and will keep throughout the year in a cool cellar.

In recovering from an illness, the system has a craving for some pleasant acid drink. This is found in cider which is placed on the fire as soon as made, and allowed to come to a boil, then cooled, put in casks, and kept in a cool cellar. Treated thus, it remains for many months as good as the day it was made.

We once saved the life of an infant which had been inadvertently drugged with laudanum, and was fast sinking into the sleep which has no awaking, by giving it strong coffee, cleared with the white of an egg, a teaspoonful every five minutes until it ceased to seem drowsy.

Our book on "Health and Disease" was written with a view to introduce people to the knowledge of items like these, in the hope of doing something towards abolishing the ruinous and almost universal habit of purchasing patent medicines, which, in so many instances, are either inapplicable, hurtful, or utterly useless, and, in this latter case, are indirectly the means of death, by the loss of time in obtaining the services of a competent physician to apply the proper means with a wise discrimination.

